# Impact of cognitive intervention on cognitive symptoms and quality of life in idiopathic Parkinson’s disease: a randomized and controlled study

**DOI:** 10.1590/1980-57642021dn15-010005

**Published:** 2021

**Authors:** Nariana Mattos Figueiredo Sousa, Ana Cristina da Mata Neri, Ivar Viana Brandi, Sonia Maria Dozzi Brucki

**Affiliations:** 1 Neurorehabilitation Program, Rede SARAH de Hospitais de Reabilitação – Reabilitação Neurológica, Unidade de Salvador – Salvador, BA, Brazil.; 2 Department of Neurology, Faculdade de Medicina, Universidade de São Paulo – São Paulo, SP, Brazil.

**Keywords:** Parkinson’s disease, cognition, rehabilitation, quality-of-life, doença de Parkinson, cognição, reabilitação, qualidade de vida

## Abstract

Pharmacological treatments for mild cognitive impairment (MCI), are lacking, and alternative approaches have been implemented, including cognitive training (CT).

**Objective:**

To determine the impact of CT on cognitive and quality of life measures in patients with Parkinson’s disease (PD) who were seen a hospital neurorehabilitation program.

**Methods:**

Thirty-nine individuals with MCI-PD, according to the Movement Disorder Society, were randomly distributed into two groups: experimental and control group, matched for demographic and clinical characteristics. Both groups were assessed for cognition and quality of life at the beginning of the study and at the end of the intervention protocol. The following instruments were used to assess cognition and quality of life: Addenbrooke’s Cognitive Examination III, Digit Span, Trail Making Test (TMT, A and B) and Parkinson disease quality of life questionnaire. The experimental group (EG) engaged in CT, whereas the control group (CG) underwent activities of the general rehabilitation program.

**Results:**

No baseline evaluation differences were found. Intergroup analysis showed differences in measures, such as total score (1.977, p=0.0480) and visuospatial domain (-2.636, p=0.0084) of the ACE-III, with the EG performing better, in addition to better performance in TMT-B mistakes (-1.928, p=0.0439). Intragroup analysis revealed that the EG showed significant improvement in almost all the cognitive variables, well as in self-reported quality of life (total score and mobility, activities of daily living, body discomfort dimensions).

**Conclusions:**

Engagement in cognitive activities was associated with better cognitive abilities in PD-MCI. Future studies should consider the long-term effect of this type of intervention and impact on functional activities.

## RESUMO.

A falta de evidência de tratamentos farmacológicos, especificamente para pacientes com comprometimento cognitivo leve na doença de Parkinson (CCL-DP), leva à implementação de abordagens alternativas, incluindo a reabilitação cognitiva.

**Objetivo:**

Determinar o impacto do treino cognitivo (TC) em medidas cognitivas e da qualidade de vida em pacientes com DP, que participavam de um programa de reabilitação neurológica hospitalar.

**Métodos:**

Total de 39 indivíduos com CCL-DP, de acordo com a Sociedade de Distúrbios do Movimento, foram distribuídos aleatoriamente em dois grupos: experimental e controle, pareados por características demográficas e clínicas. Ambos os grupos foram avaliados quanto à cognição e qualidade de vida no início do estudo e ao final do protocolo de intervenção. Os seguintes instrumentos foram utilizados para avaliar a cognição e a qualidade de vida: Exame Cognitivo III de Addenbrooke, teste de dígitos, TMT (A e B) e questionário de qualidade de vida da doença de Parkinson. O grupo experimental foi submetido ao treino cognitivo, ao passo que o grupo controle passou por atividades do programa de reabilitação.

**Resultados:**

Não foram encontradas diferenças na avaliação basal. A análise intergrupo mostrou diferenças em medidas, como escore total (1,977, p=0,0480) e domínio visuoespacial (-2,636, p=0,0084) da ACE-III, tendo o grupo experimental melhor desempenho, além de desempenho superior em TMT-B erros (-1,928, p=0,0439). A análise intragrupo revelou que o grupo experimental mostrou melhora significativa em quase todas as variáveis cognitivas, assim como na percepção de qualidade de vida (escore total e dimensões de mobilidade, atividades da vida diária e desconforto corporal).

**Conclusões:**

O envolvimento em atividades cognitivas foi associado a melhores habilidades cognitivas em pacientes com CCL-DP. Estudos futuros devem considerar o efeito a longo prazo desse tipo de intervenção e o impacto nas atividades funcionais.

## INTRODUCTION

The original description by James Parkinson mentioned a condition characterized by motor features, which included bradykinesia, tremor and gait impairment, but he also described other symptoms, without the same accuracy, such as bowel dysfunction, somnolence, delirium and constipation, which now constitute the spectrum of nonmotor symptoms (NMS) associated with Parkinson’s disease (PD).^1^ ^,^ ^2^ Such symptoms were neglected for many years but in many cases, especially in more advanced stages, may dominate the clinical picture and impair functional performance and quality of life. It is important to improve long-term outcomes by delivering therapeutic interventions earlier in the clinical course of cognitive dysfunction, although the best therapeutic decision is not precisely defined.^3^

Mild cognitive impairment (MCI) is considered an intermediate stage between normal cognition and the presence of dementia, having been initially recognized in patients with Alzheimer’s disease, but it can be present in individuals with Parkinson’s disease since diagnosis. Recommendations from the National Institute on Aging-Alzheimer’s Association workgroup defined the symptomatic pre-dementia phase of Alzheimer’s Disease and proposed a set of criteria to establish the diagnosis. Recently, the DSM 5 recognized the clinical entity of minor neurocognitive disorder (NCD) for different disorders including PD.^4^ In contrast to amnestic MCI as a prodrome to Alzheimer’s disease (AD), the Parkinson’s disease-mild cognitive impairment (PD-MCI) is more heterogeneous and may affect diverse cognitive domains. This heterogeneous clinical presentation is related to a great variety of available tests to assess cognitive functions in PD patients. The clinical diagnostic criteria were defined by the Movement Disorder Society (MDS) that proposed standardized diagnostic criteria. Routine cognitive screening is important for the optimal management of patients with PD, to assess cognition functions, and to define the diagnosis of MCI or Parkinson’s disease dementia (PDD). The Montreal Cognitive Assessment (MoCA), Mini-Mental State Examination (MMSE),^5^ and Addenbroke’s Cognitive Examination (ACE) were evaluated in different studies and populations, but even a positive screen frequently requires additional assessment through standardized neuropsychological tests, to assess the whole heterogeneity of cognitive impairment in PD patients. Patients who present deficits in semantic language, figure drawing/copying and visuospatial tasks have a higher risk to develop dementia.^6^ ^,^ ^7^

Although PD is considered a motor control disorder, deterioration in cognitive functions is a common complication, occurring in approximately 40% of cases. Half of the patients without dementia have MCI, and such changes can impair the patient’s quality of life, as well as being a risk factor for the development of dementia.^8^ Other studies report that a quarter of newly diagnosed patients already have some cognitive impairment and can affect 26.7% of patients without dementia.^9^ ^,^ ^10^ Cognitive decline, especially in executive functions, is more associated with worsening gait performance and risk of falls.^11^ Cognitive changes are characterized mainly by deficits in executive functions, visuospatial skills and attention.^12^ ^,^ ^13^

Single domain and multiple domain MCI are the most observed in this population, mainly the dysexecutive subtype; however, the criteria for defining MCI in PD are not yet fully established.^14^ Therefore, cognitive assessment and use of functional scales should be taken into account for this diagnosis. There are brief batteries that assist in the differential diagnosis of MCI and dementia in PD, such as ACE, in addition to cognitive screening instruments (such as MMSE) and standardized neuropsychological tests, to assess function cognitive.

Non-pharmacological approaches are essential for the management of cognitive symptoms, and their importance becomes even more significant in view of the lack of evidence of the effectiveness of pharmacological approaches. Recent studies have shown the effectiveness of cognitive training (CT) programs (individual or group) and cognitive-specific rehabilitation approaches have been tested in this population, due to the specificity of the neuropsychological disorder, in which impaired attention functions predominate.

Therapeutic approach in cases of PD-MCI or PDD may involve medications, such as cholinesterase inhibitors, CT, physical exercise which may include tango and/or treadmill training, brain stimulation, or combined interventions. The best therapeutic strategy is yet to be defined and the treatment of patients presenting these conditions is frequently a challenge to health professionals.^15^

There was an increase in publications on this topic not only in PD but in healthy elderly people and those with other neurological conditions.^16^ Neuroimaging studies have shown changes in activation in brain regions,^17^ ^–^ ^19^ and also a systematic review study showed that there was an improvement in global cognition and ability for planning.^20^ The studies used different assessment and intervention protocols (format of interventions and assessment instruments, sample size, follow-up time and therapeutic dose), which can interfere in the comparative and homogeneous analysis of the results. These aspects interfere in the comparison between studies and in the ability to generalize the results. Thus, there are studies with computerized CT,^21^ paper-pencil tasks^22^ and even combined with transcranial direct current stimulation.^23^ Most of these studies show that CT has a positive effect on cognitive performance and should be considered as an adjunctive therapy in PD.^24^ ^,^ ^25^

Limitations of existing research include diverse methodologies and CT programs, small samples, insufficient focus on functional outcomes, sustainability and generalization of effects of this treatment.

A study, performed in Brazil, determined the effectiveness of physiotherapy associated with CT to improve cognition and quality of life in individuals with PD, involving 58 individuals with mild to moderate PD, randomly distributed into two groups: motor group and cognitive-motor group. Intragroup analysis revealed that both groups showed improved cognition (memory and visuospatial function domains) and quality of life after execution of the protocols, but without statistically significant intergroup differences.^22^

We evaluated the therapeutic effects of non-pharmacological interventions (CT) on cognitive symptoms in PD, including control group (CG) and randomization.

The group intervention, the modality of cognitive intervention chosen for this study, allows a more direct and efficient approach to issues common to most patients, providing a moment of learning and the search for shared solutions.

Our main objective was to determine the effectiveness of a 4-week, randomized and controlled CT program in improving cognition performance and quality of life of individuals with PD.

## METHODS

This was a randomized, placebo-controlled study.

Recruitment and treatment protocol were conducted at the rehabilitation hospital from January 2019 to November 2019, at the SARAH Network of Rehabilitation Hospitals (Salvador/Bahia Unit, Brazil). A total of 39 patients (24 in the experimental group (EG) and 15 in the CG) were enrolled in this study, according to UK Parkinson’s Disease Brain Bank criteria.

### Participants and recruitment

The following inclusion criteria were applied:

- MCI according to the Movement Disorders Society (MDS) PD-MCI Level II diagnostic criteria;^23^
- presence of a stable response to antiparkinsonian medication in the pre-intervention and during the course of the intervention;
- Hoehn and Yahr (H&Y) stages I–III;
- the Beck Depression Inventory scores, with minimal to light intensity (BDI≤16); and
- not having participated in CT protocols in the year prior to enrollment.

The ACE-III battery and executive function tests (Digit Span and Trail Making Test, A and B) were used; in addition to the application of a quality-of-life questionnaire (PDQ-39), before the intervention/baseline (T0) and immediately after the intervention (T1).

The patients were divided into two groups (EG and CG), according to simple randomization. Candidate files were forbidden as to the identification and numbered in their verses from 1 to 8, after which the numerals were drawn and their respective allocation in each group: control or experimental. At the end of the allocation, professionals were made aware of the identifications to inform candidates of their respective modality of participation in the study.

The study was approved by the local Ethics Committee (CAAE: 88364618.8.0000.0022). All participants received the research details and signed on the informed consent line. Participation was carried out during “on” medication stage.

### Neuropsychological assessment

The tests were administered in a fixed order by a neuropsychologist. The following tests were administered during the “on” phase of the patients: Digit Span, TMT-A and TMT-B and short battery ACE-III.

After cognitive evaluation and application of the quality-of-life questionnaire (PDQ-39), patients who fulfilled the inclusion criteria were referred to specific groups, according to randomization.

All participants (EG and CG), during this study, participated in the general activities of the rehabilitation program for four weeks: physiotherapy, dance, reeducation in writing, speech therapy, information groups, manual skills workshops, physical activity.

### Experimental group

Participants in the EG additionally received the CT program conducted by two professional cognition experts, twice a week for 120 minutes each, totaling 8 sessions. The intervention emphasized the specific areas of cognitive deficit in this population, attention and executive dysfunction.

The CT program consisted of paper-and-pencil tasks, focused on the repeated practice of structured exercises, organized at a level of complexity and aimed to the specific cognitive domain(s), with the purpose of improving cognitive function.

Each session contemplated a function more explicitly: attention, visual memory, working memory, planning, and visuospatial and visuoconstructive abilities. The tasks involved find and mark equal figures among other similar ones, follow instructions to solve a problem, observing scenes and after evoking their details, planning and building geometrics puzzles, matching shadows between themselves focusing on the details, and reading and evoking information in a text.

The participants were asked to explore and find solutions to the initial task of each meeting, to resolve it and, after discussion with the other participants, to select the most effective strategies, being encouraged by professionals to use them for the next tasks of the same session. In the same session, three levels of difficulty were offered (easy to difficult).

### Control group

The participants participated only in the various activities of the general rehabilitation program by four weeks: physiotherapy, dance, reeducation in writing, speech therapy, information groups, manual skills workshops, and physical activity, except for activities that involved CT itself.

### Statistical analysis

Descriptive statistics (total value/percentage, mean, standard deviation and confidence interval) and inferential statistics were used. The chi-square test for nominal data and the *t*-test were used to compare demographic and clinical data between groups.

The paired-sample Student’s t-test (repeated measurement) was used to evaluate the effect of time (T0 and T1) in each group. The Student’s t-test for independent-samples was used to evaluate the effect of group (EG and CG) in T0 and T1. Correlation analyses between cognitive variables and quality of life, were performed by Spearman’s correlation.

Statistical significance was set at p<0.05. All analyses were performed using *Statistical Package for the Social Sciences* (SPSS) software, version 22.0.

## RESULTS

Twenty-four patients and fifteen controls participated in the study, matched for age, gender, education, age and disease severity, global cognition. All patients received stable drug treatment throughout this period.

The EG had a mean age of 60 (±7.5) years, and there was a higher proportion of men than women; mean years of education was 12.4 (±3.1). In relation to clinical data, patients had a mean time of disease evolution of 5.7 (±3.3) years and 87.5% were in stage I-II on the Hoehn & Yahr (H&Y) scale.

The CG had a mean age of 58.5 (±9.8) years, and there was a higher proportion of men than women; mean years of education was 12.8 (±3.4). Regarding the clinical data, patients had a mean time of disease evolution of 6.8 (±8.8) years and 93.3% were in stage I-II on H&Y (Table 1).

**Table 1:** Demographic and clinical data at baseline of patients in the control and experimental groups.

	Experimental group	(n=24) Control group (n=15)	p-value
	Mean (SD) or n (%)	Mean (SD) or n (%)	
Age, years	60.0 (7.5)	58.5 (9.8)	0.6980^a^
Gender	4 (16.67%) females and 20 (83.33%) males	2 (13.33%) females and 13 (86.66%) males	0.779^b^
ACE-III (total score)	87.5 (6.6)	87.1 (6.9)	0.2354^a^
Education, years	12.4 (3.1)	12.8 (3.4)	0.3495^a^
Duration of disease (years)	5.7 (3.3)	6.8 (8.8)	0.3309^a^
H&Y scale, n	Stage 3=3	Stage 3=1	0.6804^b^

SD: standard deviation; ACE-III: Addenbrooke’s Cognitive Examination III

a chi-square test;

b Student’s t-test for independent samples.

In Table 2, we can see that there was an improvement in measures in the post-intervention period (T1) in both groups, with better performance, in relation to the mean values for the EG.

**Table 2:** Neurocognitive performances in the control and experimental groups at baseline (T0) and retest (T1).

n=39	Control group T0 Mean (SD)	Confidence interval	Control group T1 Mean (SD)	Confidence interval
ACE-III (total score)	87.07 (7.19)	83.0872–91.0461	89.83 (6.38)	85.7800–93.8866
Attention/orientation	17.20 (1,21)	16.5315–17.8684	16.50 (1.73)	15.3995–17.6004
Memory	20.87 (4.05)	18.6233–23.1099	22.25 (4.31)	19.5121–24.9878
Verbal fluency	10.00 (1.51)	9.1627–10.8372	11.25 (1.48)	10.7837–26.0496
Language	24.93 (1.83)	23.9193–25.9472	25.42 (1.00)	24.7837–26.0496
Visuospatial function	14.07 (1.33)	13.3276–14.8057	14.42 (1.00)	13.7837–15.0496
Digit span (forward)	5.27 (0.59)	4.9379–5.5954	5.64 (0.81)	5.0928–6.1798
Digit span (backward)	3.93 (0.59)	3.6045–70.2185	4.00 (1.18)	3.2051–4.7948
Trail making test (A)	59.13 (20.02)	48.0480–70.2185	55.33 (12.94)	45.3850–65.2815
Trail making yest (B)	174.20 (122.05)	106.611–241.788	214.33 (162.58)	89.3603–339.306

n=39	Experimental group T0 Mean (SD)	Confidence interval	Experimental group T1 Mean (SD)	Confidence interval
ACE-III (total score)	87.50 (6.76)	84.6442–90.3557	92.26 (5.17)	90.0244–94.4973
Attention/orientation	16.71 (1.46)	16.0922–17.3244	17.13 (1.25)	16.5880–17.6728
Memory	20.75 (3.74)	19.1688–22.3311	22.96 (2.62)	21.8237–24.0892
Verbal fluency	9.92 (2.36)	8.92116–10.9121	11.61 (3.77)	9.9763–13.2410
Language	25.54 (0.72)	25.2371–25.8461	25.22 (3.33)	23.7775–26.6572
Visuospatial function	14.58 (1.50)	13.9494–15.2172	15.35 (1.07)	14.8848–15.8108
Digit span (forward)	5.33 (1.20)	4.82498–5.8416	5.65 (0,98)	5.2275–6.0768
Digit span (backward)	3.83 (0.76)	3.51182–4.15483	3.91 (1.08)	3.4445–4.3815
Trail making test (A)	63.71 (19.57)	55.4426–71.9740	61.52 (25.38)	50.5456–72.49781
Trail making test (B)	193.38 (126.62)	139.909–246.8403	188.74 (93.14)	148.4612–229.0171

Mean (standard deviation [SD]); ACE-III: Addenbrooke’s Cognitive Examination III.

As seen in Table 3, the baseline evaluation (T0) did not show any difference in the cognitive variables between the groups. In the T1 evaluation, there were differences in measures, including total score (1.977, p=0.0480^*^) and visuospatial domain (-2.636, p=0.0084^*^) in the ACE-III, with the EG performing better, in addition to better performance in TMT-B mistakes (-1.928, p=0.0439^*^).

**Table 3:** Inferential analysis — experimental x control.

Cognitive tasks	Baseline (T0)		Post-intervention (T1)	
	t-test	p-value	t-test	p-value
Digit span forward	-0.456	0.6487	-0.039	0.9685
Digit span backward	0.360	0.7191	0.268	0.7887
TRAIL A second	-0.867	0.3862	-0.482	0.6297
TRAIL A mistakes	1.265	0.2059	-0.626	0.5316
TRAIL B second	-1.039	0.2987	-0.231	0.8177
TRAIL B mistakes	1.634	0.1022	-1.928	0.0439*
Total score (ACE-III)	-1.187	0.2354	1.977	0.0480*
Attention/orientation	-1.072	0.2838	-1.327	0.1844
Memory	0.174	0.8616	0.000	1.0000
Verbal fluency	-0.117	0.9069	0.177	0.8594
Language	-1.150	0.2503	-1.395	0.1631
Visuospatial function	-1.276	0.2018	-2.636	0.0084*

Student’s t-test for independent samples (group effect);

* p<0.05;

** p<0.01.

To compare and measure the effect of the intervention, in relation to the EG, parametric statistics were used paired-sample Student’s t-test after normality analysis (Shapiro-Wilk p>0.05). Table 4 presents the results showing improvement in attention/orientation subscores (-2.228, p=0.0259^*^), memory (-3.221, p=0.0013^*^), verbal fluency (-2.133, p=0.0329^*^) and visuospatial function (-2.562, p=0.0104^*^), in addition to the total score (-3.686, p=0.0002^*^) in the ACE-III battery. Regarding the standardized neuropsychological tests, improvement was observed in tests that evaluate alternate attention and visuomotor processing speed (TMT-B errors -1.646, p=0.0398^*^; TMT-A seconds -0.700, p=0.0484^*^). In the CG, improvement was observed in domains including verbal fluency (-2.020, p=0.0434^*^) and visuospatial function (-2.227, p=0.0260^*^) in the ACE-III.

**Table 4:** Inferential analysis — baseline x post-Intervention evaluations.

Cognitive tasks	Control group		Experimental group	
	t-test	p-value	t-test	p-value
Digit span forward	-1.414	0.1573	-1.384	0.1664
Digit span backward	0.000	1.0000	-0.113	0.9103
TRAIL A second	-0.178	0.8590	0.700	0.0484*
TRAIL A mistake	1.000	0.3173	-1.000	0.3173
TRAIL B second	-0.059	0.9528	-0.532	0.5945
TRAIL B mistake	0.914	0.3609	1.646	0.0398*
Total score (ACE-III)	-0.029	0.9769	-3.686	0.0002*
Attention/orientation	1.386	0.1657	-2.228	0.0259*
Memory	-1.327	0.1844	-3.221	0.0013*
Verbal fluency	-2.020	0.0434*	-2.133	0.0329*
Language	-1.379	0.1677	-1.335	0.1819
Visuospatial function	-2.227	0.0260*	-2.562	0.0104*

Paired-sample *t* test (baseline and post-intervention) in each group (intragroup analysis);

* p<0.05;

** p<0.01.

Regarding quality of life (PDQ-39 values), there was an improvement in the total score, mobility, activities of daily living and body discomfort dimensions in the EG. The CG showed improvement only in the total score (Table 5).

**Table 5:** Inferential analysis — baseline x post-intervention evaluations.

PDQ-39	Control group		Experimental group	
	t-test	p-value	t-test	p-value
Total score	1.889	0.0588*	2.275	0.0229*
Mobility	1.916	0.0554	2.680	0.0074**
Activities of daily living	0.905	0.3656	3.317	0.0009**
Emotional well-being	1.560	0.1188	1.326	0.1848
Stigma	1.882	0.0599	0.335	0.7379
Social support	-0.771	0.4406	-0.957	0.3386
Cognition	0.884	0.3768	0.405	0.6853
Communication	0.159	0.8733	-0.217	0.8283
Body discomfort	0.666	0.5057	2.847	0.0044**

Paired-sample *t* test (baseline and post-intervention);

* p<0.05;

** p<0.01.

Correlations between quality-of-life data (PDQ-39) and cognitive scores showed significant interaction between the total score of the PDQ-39 questionnaire and TMT-B (r=0.3724, p=0.0358) and activities of daily living of PQD-39 and TMT-B (r=0.4453, p=0.0106) in the post-intervention group.

No adverse effects were reported during treatment in either group.

## DISCUSSION

Cognitive dysfunctions are common non-motor symptoms in PD and are generally associated with a worse prognosis. The current study aimed to evaluate cognitive intervention (CT) in cognitive and quality of life measures in patients with PD-MCI.

The current intervention program, in a group format, was beneficial in these patients.

The results showed improvement, after this intervention program in aspects, mainly attention (especially test of shifting attention and processing speed), executive (verbal fluency) and global measures in the ACE-III battery, in agreement with other studies that have shown the benefits of this type of intervention in Parkinson’s patients.

Regarding quality of life, the results showed a significant improvement in the total score and in the dimensions of mobility, activities of daily living and body discomfort. The results, therefore, are in line with data from the literature demonstrating that cognitive interventions are effective in patients with PD.^24^ ^–^ ^26^

In the CG, improvement was observed in the domains verbal fluency (-2.020, p=0.0434^*^) and visuospatial function (-2.227, p=0.0260^*^) in the ACE-III (Table 2), as was also observed in the CG, but with a slightly lower level of significance.

The CG also showed improvement after CT, but the intervention group showed improvement in more cognitive and quality of life measures. It is worth mentioning that the groups were homogeneous in demographic and clinical aspects, making the analysis of the intervention effect with less bias, such as interference from the learning effect.

Despite the PDQ-39 instrument being the most used in this population, we observed few items that assess the cognitive dimension, while others have a higher quantity. These aspects may have interfered with the results observed in this study.

The maintenance of the effects of CT over time has also been the object of investigation. However, there are few studies that maintain longitudinal monitoring.^27^ A study that maintained longitudinal monitoring for 3 months after the intervention observed persistent results in language, attention and executive functions.^28^ In turn, the maintenance of benefits after one year of participation in a structured and consistent training program was observed.^10^

Studies that contemplate a more systematic follow-up may help in the design of training programs more useful to this population.

The improvement observed also in the CG could be attributed to the benefit that motor activity can have for cognition, since the general activities of the rehabilitation program carried out in both groups have a greater character of motor training, and as its execution demands cognitive skills (attention and executive functions), it is possible that they offer cognitive challenges to individuals with PD and this has also been reflected in the CG.^22^

The methodological variability of the training programs established in the studies can include sample size, therapeutic dose (number and duration of intervention sessions), evaluation and intervention protocols, absence of follow-up and variable follow-up times, which are some of the aspects that hinder the more precise definition of the effects of CT and which demand more and more investigations in this area. Moreover, most of the studies included a cognitively mixed PD group in terms of cognitive decline; these studies might have less ability to detect treatment effects, as ceiling effects may play a role in cognitively unimpaired individuals.

Despite the methodological improvement of studies with group CT, it is difficult to compare them and generalize the results.

This study, even though on a small study sample, showed differential treatment effects for a PD-MCI group.

The results of our study showed the importance of early cognitive assessment and intervention in the PD-MCI population. There is a greater benefit and effectiveness of the CT as they have more preserved skills. Most of our sample consisted of patients who were still working or had an active routine of activities. In this study, however, it was not possible to assess the persistence of cognitive gain after training (follow-up), and only the assessment was carried out immediately after the intervention. However, the demographic and clinical homogeneity between the groups (EG and CG) at baseline (T0) contributed to the analysis of the effectiveness of cognitive intervention.

The current study had as strengths:

- neuropsychological assessment with global and cognitive and sensitive and standardized assessment tests for this population;
- groups with homogeneous profile;
- systematization and standardization of the mediation/intervention process; and
- CG matched by demographic and clinical characteristics.

The limitations were:

- the progressive characteristic of the pathology increases the risk of cognitive worsening and development of dementia, which may influence the results of the reassessment;
- the reduced intensity of interventions (therapeutic dose) of cognitive training when compared with previous studies on neuropsychological intervention; and
- absence of follow-up.

Although more controlled studies are needed to demonstrate the effectiveness of CT interventions, the current study did highlight that a CT treatment can be useful to improve cognitive functioning in PD patients. Future studies should consider the long-term effect of this type of intervention and impact on functional activities of this treatment. These findings support the integration of CT into the standard of care for patients with PD.
